# Feasibility and Acceptability of A Self-Directed Virtual Interview Preparation Tool for Medical Students

**DOI:** 10.1007/s40670-025-02627-x

**Published:** 2026-03-11

**Authors:** Anthony L. Shanks, Ella Boardley, Avery Dawes, Ashley Holt, Debra Rusk, Emily Walvoord

**Affiliations:** 1 Indiana University School of Medicine, Indianapolis, IN USA; 2 Ascension Saint Vincent Hospital, Indianapolis, IN USA

**Keywords:** Artificial intelligence (AI), Residency interviews, The match, Fourth year medical school curriculum, Interview preparation, Virtual interviews

## Abstract

**Background:**

The conversion to virtual interviews holds many benefits. However, lack of familiarity with the format may put residency applicants at a disadvantage.

**Objective:**

To describe our experience after implementing a web-based system that allow students to rehearse virtual interviews and receive feedback.

**Methods:**

Access to a virtual interview practice platform (VIPP) was provided to all students in the Indiana University School of Medicine Class of 2023. Use of the program was encouraged but not mandatory. The most utilized questions and most missed questions were reported. A cross-sectional survey was conducted via Qualtrics to assess student satisfaction. Descriptive statistics were performed using SPSS 29.0 and Chat GPT was used for thematic analysis.

**Results:**

During the 2022-2023 year, 214 out of 365 students (58.6%) utilized the VIPP, and 29 students completed the follow up survey. The most difficult questions were: “tell me about a failure”, “tell me about a mistake you made and what you learned” and “discuss a challenging patient.” Thematic analysis of survey responses identified three main themes: positive impact on interview preparation, mixed feedback on effectiveness and customization needs, and suggestions for improvement. Of those surveyed, 97% (N = 28) would recommend VIPP to students and the majority (N = 27, 93%) agreed that the resource prepared them for virtual interviews.

**Conclusions:**

Students who used the VIPP felt the program prepared them well for virtual residency interviews. Given the importance of the interview in the residency application process, integration of this resource could assist students obtaining valuable interview practices.

**Supplementary Information:**

The online version contains supplementary material available at 10.1007/s40670-025-02627-x.

## Background

Interviews are a key component in the residency selection process. Interviews allow program directors to evaluate the compatibility between applicants and the residency program to ensure a good match in terms of values and culture. The interview further allows for evaluation of critical thinking skills, resilience, and life experience of applicants. Program directors consistently rate a student’s interactions with faculty during the interview process as one of the most important factors in a student’s ranking for the Match [1, 2].

Mock interviews play a crucial role in student preparation as they provide realistic practice and the opportunity to receive constructive feedback [3]. Interview practice can also provide a chance for self-reflection, allowing students to improve their confidence and refine their responses [4]. Following the COVID-19 pandemic, there has been widespread adoption of virtual interviews [5]. Despite the advantages of virtual formats, students cite significant anxiety regarding preparedness for a virtual format and program perception of the interviewee in these settings [3, 6]. Students with more self-reported anxiety perform worse on virtual interviews than those with reportedly less anxiety, and anxiety levels have been noted to be higher for virtual interviews than for in-person interviews [7]. Though virtual interview preparation guides exist, the impact of formal training programs is unknown [8]. New technology takes adaptation, and attitudes are a major link in predicting whether students intend to use new technology. Perceived usefulness, ease of use, and risk all influenced acceptance by shaping those attitudes [9].

The lack of opportunity to practice interview skills may contribute to increased anxiety [3]. As interviews have moved to a virtual format, the ability to practice virtual interview skills, rather than in person skills, is of specific importance [5]. In 2022, Indiana University School of Medicine (IUSM) purchased access to a virtual interview practice platform (VIPP). The VIPP (BIG Interview Medical) is a commercially available resource that utilizes a web-based system allowing students to practice virtual interviews. The program contains hundreds of interviews, questions, and sets for student practice. Using artificial intelligence (AI), the program provides feedback on video recordings and written lessons. The objective of our study is to demonstrate the acceptability and feasibility of implementing a commercially available self-directed online virtual interview preparation tool provided free-of-charge to a class of medical students in the fourth year of medical school.

## Methods

Access to the VIPP was provided free of charge to all students in the Class of 2023 at IUSM.

Upon logging in, students are presented with a dashboard that allows them to select prerecorded video or written lessons based on their preferred learning format. These lessons cover the same material regardless of format. Self-directed topics include interview basics, commonly asked questions in an interview, and behavioral and personality questions. Guidance on answering questions related to challenges such as not matching in a previous cycle was also provided. Students could work at their own pace and select general interview questions to practice as well as specialty-specific questions. Students used their web camera to record their responses, and the program provided feedback on the answer length, body language, eye contact, and verbal ticks. There was also the option to answer questions in a written format without a web camera.

Recorded videos were assigned a grade out of 100 and shown on the student dashboard. A token was affiliated with each score with high scores receiving a gold, middle scores silver, and lower scores bronze. By clicking on the token, students were able to see feedback via the AI component on pace of speech, the use of power words, and eye contact with the web camera. A “power” word is a term used to describe words that evoke strong emotions, create vivid imagery, or inspire action [10]. These words are often used in marketing, writing, and speeches to grab attention and persuade the audience [10]. Examples of power words include “amazing,” “unbelievable,” “transform,” “discover,” and “ultimate.”

Use of the service was encouraged but not mandatory. Following the program’s use, medical school leadership was able to access the dashboard to evaluate student performance. Additionally, a cross-sectional survey was given via Qualtrics to assess satisfaction with the program after the Residency Match in April of 2023. Survey items were developed through an iterative process via Medial Student Affairs. Meetings were held to discuss the virtual interview service and to identify relevant questions with refinement of phrasing to best capture intended responses. The survey included Likert responses as well as the ability to provide free text feedback on the service. Descriptive statistics were performed to summarize usage data from the virtual interview platform with SPSS 29.0. This included calculation of frequencies, percentages, means, and standard deviations for variables, such as the number of lessons reviewed. Qualitative analysis of free text was evaluated with ChatGPT and the following zero-shot prompt [11], “Analyze the following free-text comments from our survey and group them into themes.” The AI-initiated analysis facilitated the identification of recurring patterns within the qualitative data and clustered them. Following the use of ChatGPT, responses were assessed for authenticity and accuracy by the authors. The study was deemed exempt by IUSM IRB (#16541).

## Results

Two-hundred fourteen out of three hundred sixty-five medical students (58.6%) in the class of 2023 accessed the VIPP and both video and written lesson formats were utilized. The average number of written lessons reviewed per student was 10 (max 24, SD 9.1). The average number of video lessons reviewed per student was 16 (max 24, SD 9.0).

Answers to practice interview questions are scored 0–100, with higher scores indicating better student performance on a given question, while lower scores represent poorer performance. Analysis of recorded responses revealed that the most difficult questions based on student scores were: “tell me about a failure” (average score 55), “tell me about a mistake you made and what you learned” (average score 58), “tell me about one of the most stressful experiences you had during medical school” (average score 61), “tell me about one of your most challenging patients” (average score 61), and “tell me about a time when you had too much to do and missed a deadline” (average score 62).

**Fig. 1:**
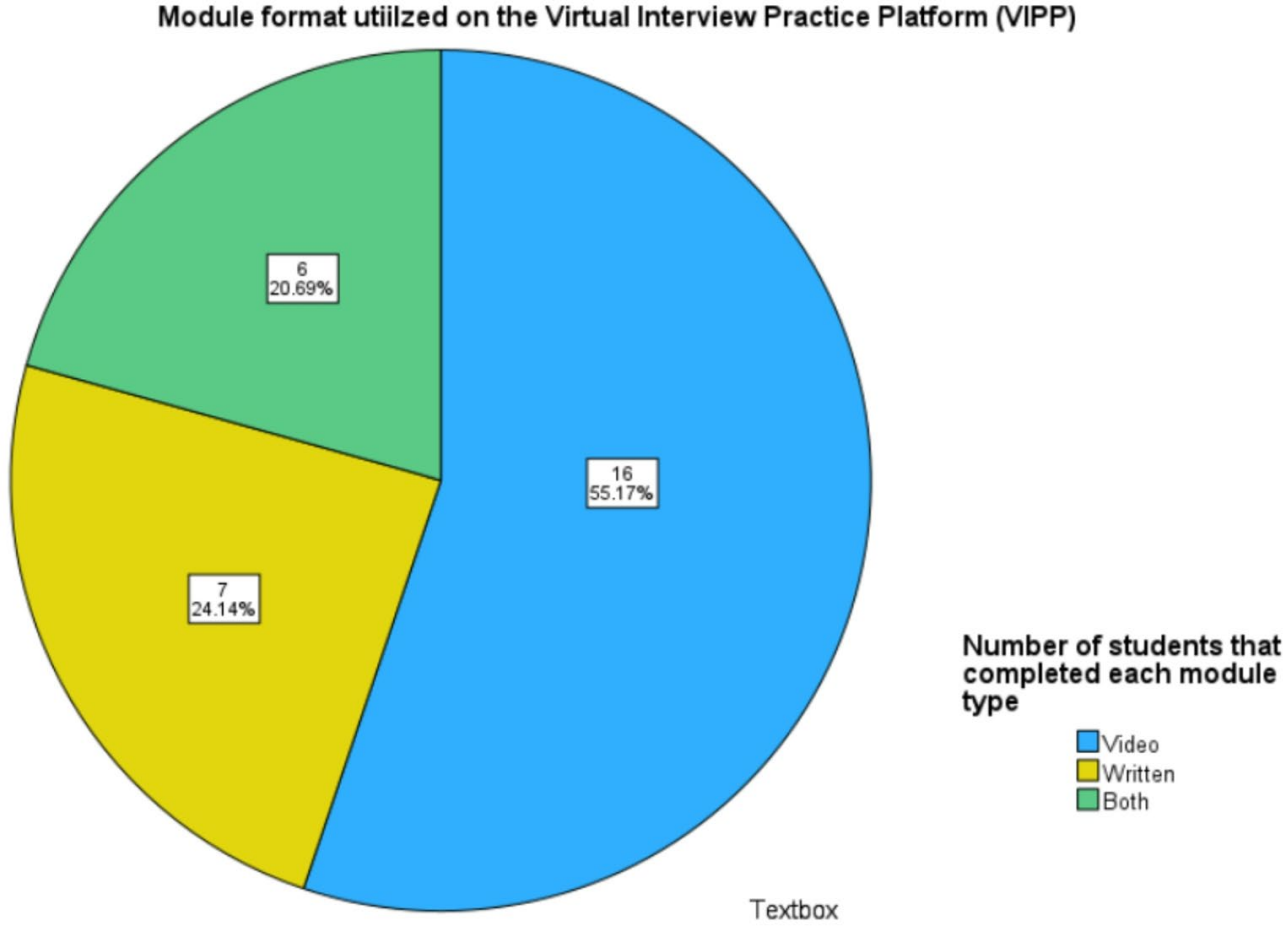
Types of BIG interview medical modules completed by survey respondents

Twenty-nine students responded to our survey for post-implementation feedback. Students who only accessed written lessons answered only the survey questions pertaining to written lessons and left those regarding the video portions of the AI tool blank, and vice versa. Video modules were the most utilized with 22 of 29 respondents accessing this type of instructional format (Fig. 1).

**Fig. 2:**
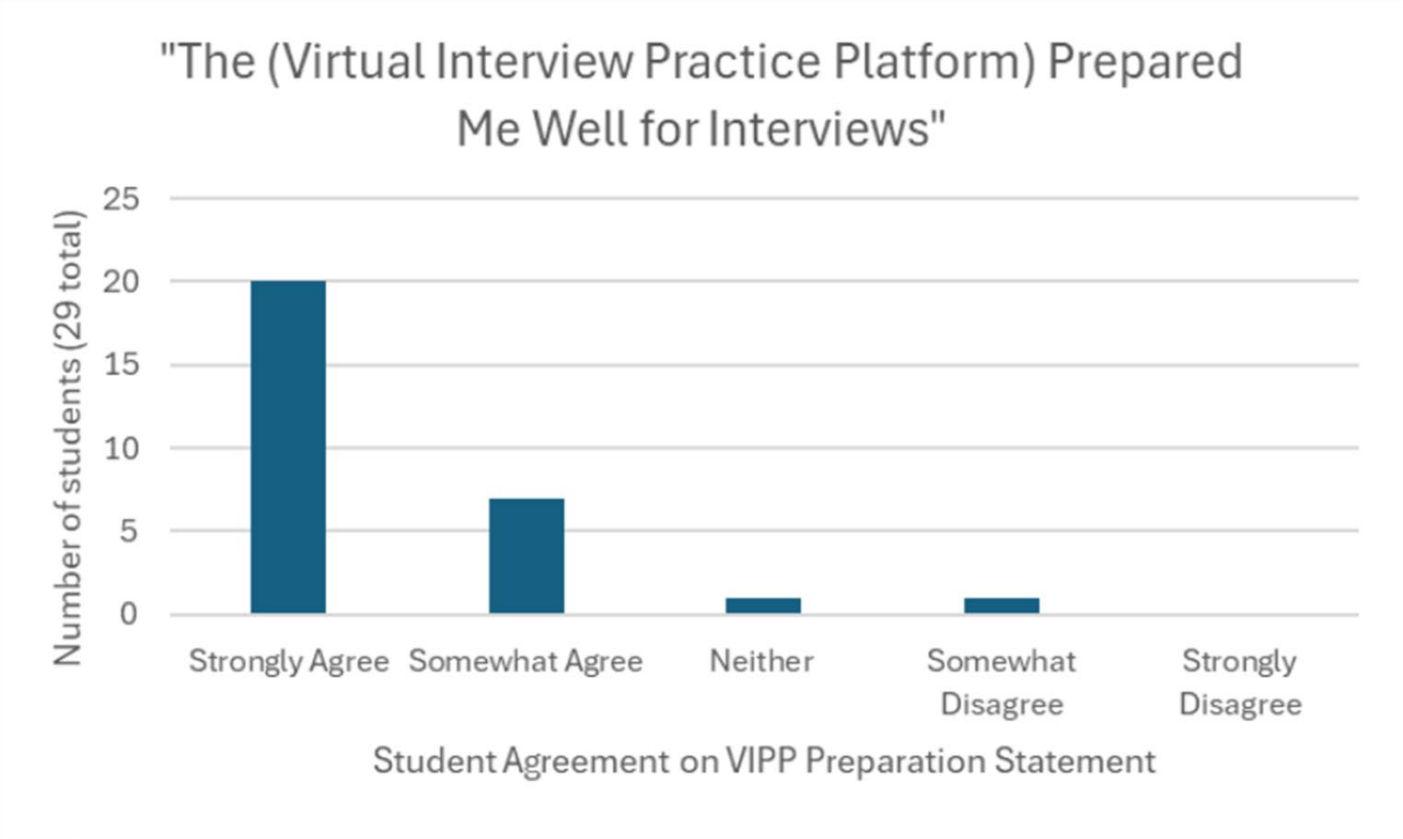
Student opinion on BIG interview medical program

Overall, students were satisfied with the resource with 97% of respondents (N = 28) recommending the use of the VIPP to future students. Almost all students that responded to our survey (27/29, 93%) agreed with the statement: “BIG Interview Medical prepared me well for interviews” (Fig. 2).

Students were also asked what other tools they used to prepare themselves for virtual residency interviews. Many students (*N* = 16, 55.2%) used other resources for practice interview questions besides the VIPP and mock interviews which included YouTube, Google, and Reddit. Most respondents (*N* = 25, 86%) would recommend the VIPP for future medical students.

Thematic analysis of free text responses identified three main themes: positive impact on interview preparation, mixed feedback on effectiveness and customization needs, and criticisms and suggestions for improvement (Table 1). Positive impact on interview preparation was supported by student responses including “amazing resource that made me feel prepared for interviews” and “I used the examples from BIG Interview to then draft my own answers to commonly asked questions.” The theme of mixed feedback on effectiveness and customization needs was identified based on comments such as “I would have liked a more formal option to practice interviewing with faculty who could give more feedback than BIG Interview Medical” and “what was the most useful for me was the lists of common questions and guides on how to create answers to some of the most common questions.” Lastly, criticisms and suggestions for improvement included comments from students such as “much of the advice from BIG Interview Medical did not apply to (my specialty)” and “BIG Interview Medical only made me more stressed.”

**Table 1.**
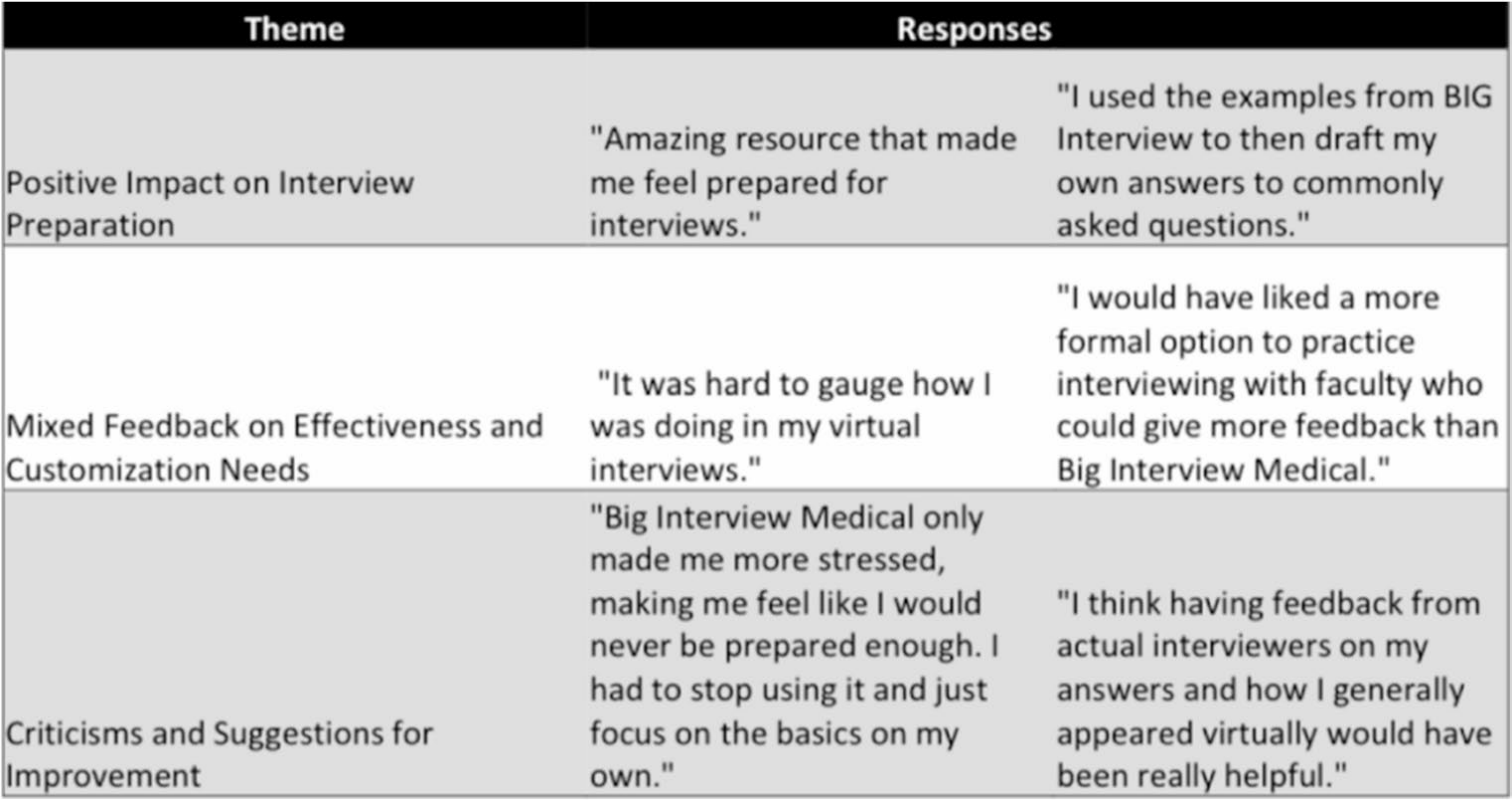
Thematic analysis of free text responses

## Discussion

Students who utilized the VIPP felt the program prepared them well for virtual residency interviews, and the majority would recommend the use of the VIPP to future classes in their preparation. Given the importance of the interview during the residency application process, relevant and practical tools are needed to ensure student success. The results of this study indicate that the use of the VIPP is a potentially useful tool in preparing fourth-year medical students for virtual interviews for residency.

Interviews can generate significant anxiety for students, and practice is one way to mitigate it [12]. Students who participate in practice interviews feel better prepared for authentic interviews, more confident, and believe the practice helps improve their skills [4, 13]. Having the ability to practice interviews in a video format would be beneficial in today’s virtual interview climate [14]. Research has shown that in preparation for virtual interviews, students require the most help in their preparation with the delivery of content and video technical quality [15].

Mock interviews can offer feedback in these specific areas, which allows students to make adjustments prior to official interview season. Addressing concerns such as video quality can not only aid in preparation for interviews but can also help students present themselves appropriately for virtual open houses and “Zoom happy hours” which many programs have adopted [16]. Additionally, mock interviews can allow students to identify commonly asked questions and work on their answers. At our institution, mock interviews were inconsistently performed and lacked standardization. VIPP offers a more uniform and accessible format, though its effectiveness compared to traditional mock interviews remains uncertain. Comparing virtual mock interviews to in-person mock interviews is an area of future study.

Compared to in-person mock interviews, VIPP offers scalable, flexible, and self-directed practice with automated feedback. In addition, the VIPP can add to this benefit by allowing advisors to track what questions were the most troublesome for their students. Students in our study had the most difficulty answering questions relating to personal failure, mistakes, stressful experiences, challenging patients, and missed deadlines. Though the scoring from VIPP is not transparent it highlights specific coaching points for students in preparation for interviews. Having the opportunity to practice and receive feedback regarding their responses to these questions may assist them in preparing for real interviews.

It is known that students often seek out information outside of school-sanctioned venues. Survey respondents indicated that the resources they used in addition to the VIPP included YouTube, Reddit, and Google. These resources lack transparency about what questions students are viewing and do not provide active feedback as to how they perform on questions.

The use of VIPP as a tool allows students to evaluate their performance with the virtual format and provides medical schools with the opportunity to evaluate areas for improvement. While the intent of the VIPP is to alleviate student anxiety regarding virtual interview preparation, we found it interesting that one respondent to our survey felt more stress when using the program. This may be worthy of further research as the program is integrated into medical schools and advising plans.

Integration of rapidly developing systems, such as AI, requires thoughtful deployment. As technology continues to evolve exponentially, it is important to be mindful of its adoption into medical education. One consideration that was emphasized by student respondents was the desire for more personalized feedback. Similarly, a study amongst pharmacy students also felt the limitation of authenticity with VIPP [13]. The use of new technologies invariably requires assistance and guidance on usage. The future use of the program may be most beneficial as an adjunct to personalized feedback with lead advisors or specialty mentors rather than being a replacement for these tools.

Strengths of the study include its large sample size of students who utilized the program. IUSM is the largest allopathic medical school in the United States, and our results therefore may be generalizable to a larger population [17]. Furthermore, the program was provided to all students free of cost, minimizing inequities between students. The web-based format of the program allows students from across the state to participate and practice irrespective of their geographic location or clerkship assignment, further expanding the generalizability of the results. The use of both quantitative and qualitative data adds to the strength of the study and future implications.

Our study is not without limitations. Though access was provided to the entire school, not all students took advantage of the resource. As a result, the findings may disproportionately reflect the views of students who were more motivated or interested in responding. One of the primary concerns regarding new technology in the medical education space is the potential to exacerbate inequities. To minimize this in our study, it was important to provide resources to all students free of charge. Additionally, while survey responses were overwhelmingly positive, our low survey response rate (14%, *N* = 29) introduces selection bias and may limit its generalizability.

In our methodology, ChatGPT was used to summarize themes in the data. ChatGPT can function as a valuable tool during analysis, enhancing the efficiency of the thematic analysis and offering additional insights into the qualitative data. The benefits of thematic summary are convenience and speed. It is not without limitations though. There can be loss of nuances, risk of bias, and lower interpretive depth [18].

In addition, responses were voluntary. This is a single-site study, which remains an inherent limit to generalizability outside of a single institution. In addition, the VIPP scoring rubric is not transparent, which limits the interpretation of the performance data. While students felt that the scoring provided by the AI was helpful, there was a lack of clarity in how the score is derived. It may be best to use VIPP in addition to feedback from other mentors and advisors. Students are still encouraged to click the token for formative feedback on their virtual interview performance. Lastly, we do not have real-world outcome data as our study did not provide information about individual performances in actual residency interviews in comparison to VIPP practice. It is unknown whether increased usage of the program is correlated with improved interview performance or match ranking.

## Conclusions

While multiple factors contribute to a successful residency match, residency interviews carry significant importance and adequate preparation with available tools may be helpful to student success. Our findings suggest that VIPP has the potential to support students though its ultimate value should be confirmed by future studies which assess interview performance and match outcomes. Survey responses indicate that students felt that VIPP can augment interview preparation and would recommend future students to use the service. Medical schools can use this information to help better prepare graduating students for a successful interview process and subsequent Match. Future directions involve comparing VIPP to in-person mock interviews, assessing cost-effectiveness and determining how its use correlates with match outcomes.

## Supplementary Information

Below is the link to the electronic supplementary material.

Supplementary File 1(DOCX 410 KB)
