# Supplementary material for: Feasibility and Acceptability of A Self-Directed Virtual Interview Preparation Tool for Medical Students
Source: Med Sci Educ. 2026 Mar 11;36(3):1533–8. doi: 10.1007/s40670-025-02627-x (PMC13355977; doi:10.1007/s40670-025-02627-x)
Supplement: Supplementary file 1 — (DOCX 410 KB) [file 40670_2025_2627_MOESM1_ESM.pdf]

Default Report

Big Interview Medical

August 13, 2025 7:27 AM MDT

Q_RecaptchaScore

#	Field	Minimum	Maximum	Mean	Std Deviation	Variance	Count
1	Q_RecaptchaScore	0.80	1.00	0.96	0.06	0.00	40

Q1 - The Big Interview Medical Curriculum is offered in both written and video formats.

Please select below which you completed.

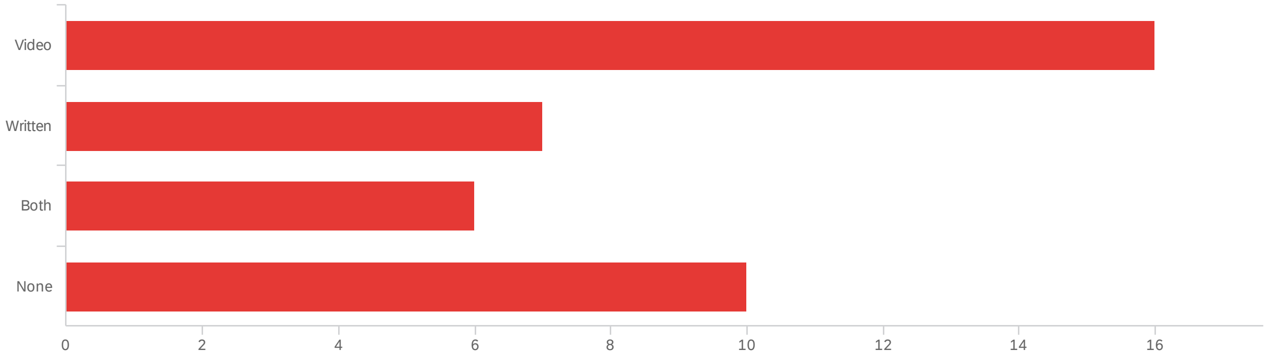

#	Field	Minimum	Maximum	Mean	Std Deviation	Variance	Count
1	The Big Interview Medical Curriculum is offered in both written and video formats. Please select below which you completed.	1.00	4.00	2.26	1.23	1.52	39

#	Field	Choice Count	
1	Video	41.03%	16
2	Written	17.95%	7
3	Both	15.38%	6
4	None	25.64%	10
			39

Showing rows 1 - 5 of 5

Q12 - Of the lessons you reviewed, please select the number in each category that you completed.

#	Field	Minimum	Maximum	Mean	Std Deviation	Variance	Count
1	Written	0.00	24.00	9.94	8.85	78.41	17
2	Video	0.00	24.00	14.88	8.82	77.87	25

Q2 - Big Interview provides the opportunity to practice what you have learned from the curiculum and save recorded video responses. How many videos did you record?

Big Interview provides the opportunity to practice what you have learned fr...
0
None
0
0
None
0
0
13
0
Probably around 50
8+
0
0
none
0
3-4
5-6
47
0
0
2

Big Interview provides the opportunity to practice what you have learned fr...
8
0
2
20
0
0
8
0
8
0
None

Q4 - Big Interview Medical prepared me well for interviews.

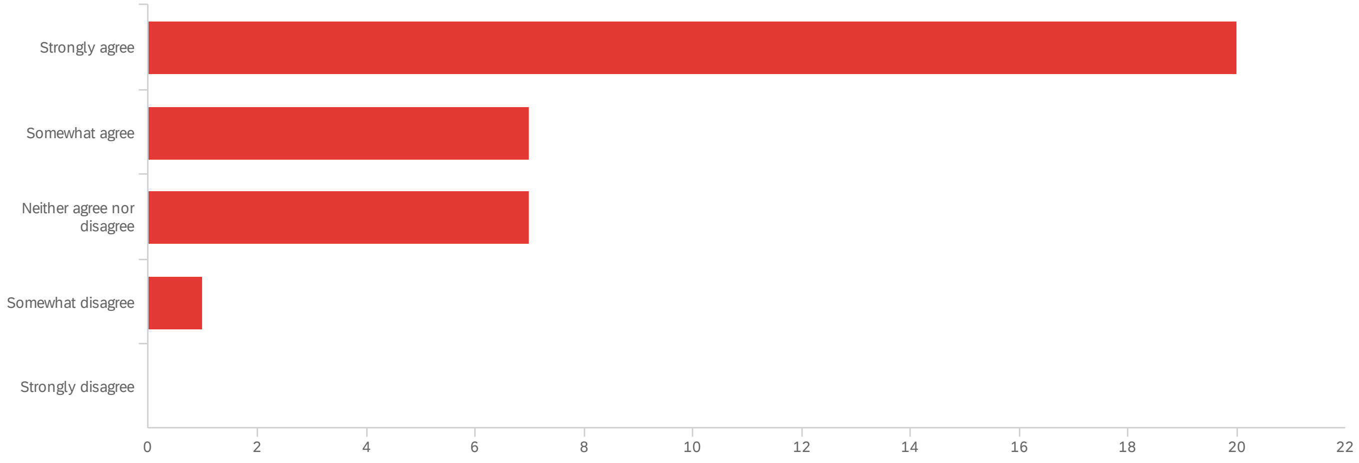

#	Field	Minimum	Maximum	Mean	Std Deviation	Variance	Count
1	Big Interview Medical prepared me well for interviews.	6.00	9.00	6.69	0.89	0.79	35

#	Field	Choice Count	
6	Strongly agree	57.14%	20
7	Somewhat agree	20.00%	7
8	Neither agree nor disagree	20.00%	7
9	Somewhat disagree	2.86%	1
10	Strongly disagree	0.00%	0
			35

Showing rows 1 - 6 of 6

Q5 - The AI feedback on my recorded videos helped me identify areas of weakness

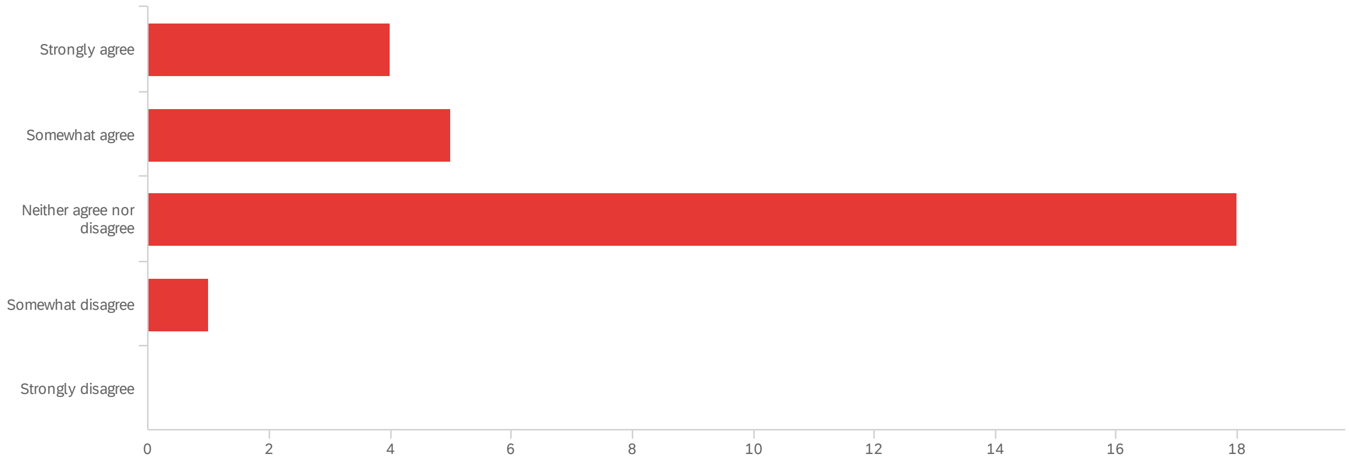

#	Field	Minimum	Maximum	Mean	Std Deviation	Variance	Count
1	The AI feedback on my recorded videos helped me identify areas of weakness	6.00	9.00	7.57	0.78	0.60	28

#	Field	Choice Count	
6	Strongly agree	14.29%	4
7	Somewhat agree	17.86%	5
8	Neither agree nor disagree	64.29%	18
9	Somewhat disagree	3.57%	1
10	Strongly disagree	0.00%	0
			28

Showing rows 1 - 6 of 6

Q6 - Big Interview Medical made me feel more comfortable with virtual interviews

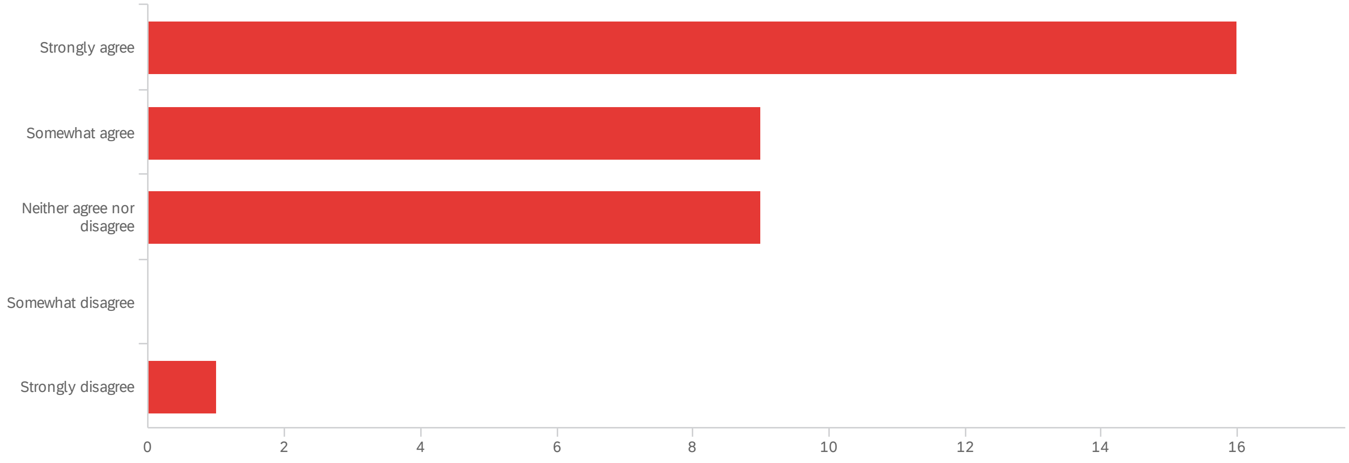

#	Field	Minimum	Maximum	Mean	Std Deviation	Variance	Count
1	Big Interview Medical made me feel more comfortable with virtual interviews	6.00	10.00	6.89	0.98	0.96	35

#	Field	Choice Count	
6	Strongly agree	45.71%	16
7	Somewhat agree	25.71%	9
8	Neither agree nor disagree	25.71%	9
9	Somewhat disagree	0.00%	0
10	Strongly disagree	2.86%	1
			35

Showing rows 1 - 6 of 6

Q7 - I used resources other than Big Interview Medical to prepare for my residency interviews

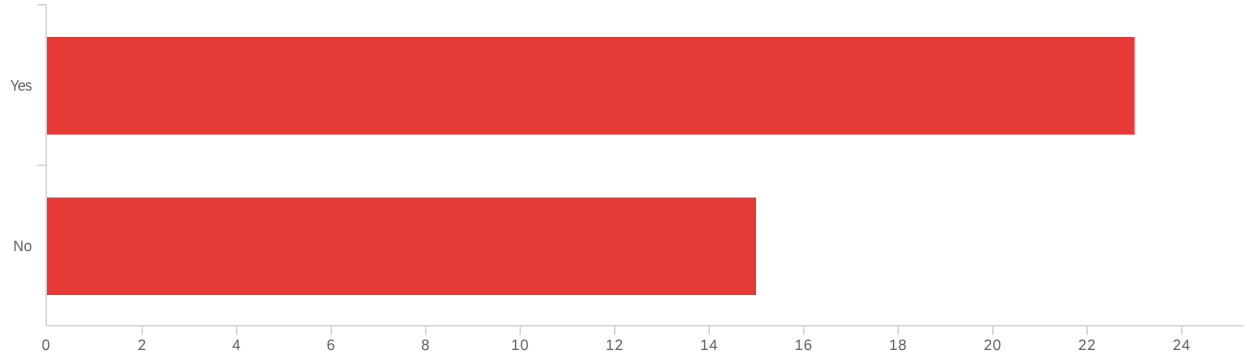

#	Field	Minimum	Maximum	Mean	Std Deviation	Variance	Count
1	I used resources other than Big Interview Medical to prepare for my residency interviews	1.00	2.00	1.39	0.49	0.24	38

#	Field		Choice Count
1	Yes	60.53%	23
2	No	39.47%	15
			38

Showing rows 1 - 3 of 3

Q8 - What additional resources did you use? Please list all

What additional resources did you use? Please list all
random websites on google. IM google doc with interview questions (generally public somewhere online)
People in my life
Forums, YouTube
AMSER Guide to Applying for Radiology Residency
None
I just Googled for interview resources, which may not have been the best idea, but there were some good free resources out there.
Google Reddit list of common questions I found
Reddit, other online forums
IU provided resources and multiple other forums/articles/blogs on the internet.
Career mentor
Reddit
friends and family
Googled to look at some good answers to common questions
Youtube videos on common residency interview questions and helpful tips for interviews.
Mock interview with faculty Reddit list of possible interview questions
Google searches, mentors

Q9 - I would recommend Big Interview Medical to future medical students

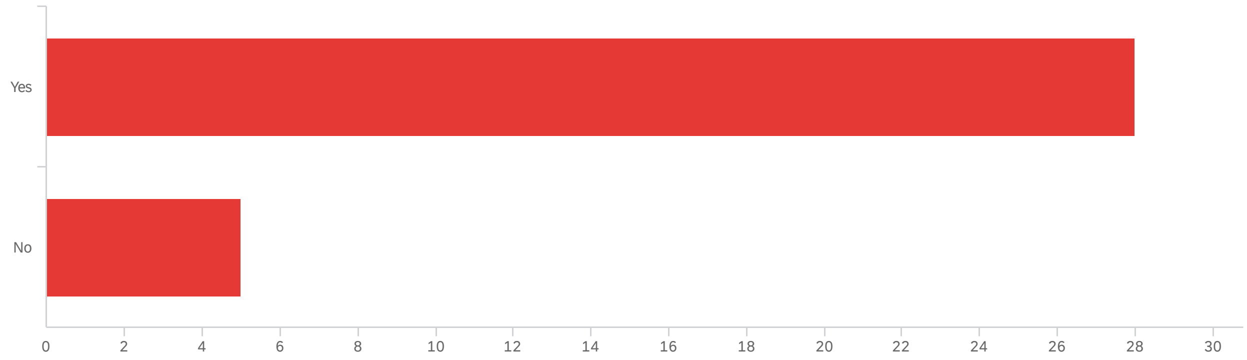

#	Field	Minimum	Maximum	Mean	Std Deviation	Variance	Count
1	I would recommend Big Interview Medical to future medical students	1.00	2.00	1.15	0.36	0.13	33

#	Field	Choice Count	
1	Yes	84.85%	28
2	No	15.15%	5
			33

Showing rows 1 - 3 of 3

Q10 - Do you have any additional recommendations regarding usage or implementation to share?

Do you have any additional recommendations regarding usage or implementatio...
No
I used the examples from Big Interview to then draft my own answers to commonly asked questions
Practice practice practice!
No, very intuitive
There are a lot of different opinions regarding how to prepare for a residency interview out there, and I think Big Interview Medical does a good job of helping make the initial framework as of what information might be good to try to include. I would not necessarily follow all of Big Interview Medical's recommendations as they are written.
Probably a great program but I didn't use it. It felt extremely unnatural and I could get past it
I used the program to help come up with answers to common interview questions but did not use the interview prep recording features. I found it helpful to think about how I might answer certain questions so for that reason would recommend it.
It was a good starting place, especially with the amount of possible questions they had. That was what I mainly used it for. The AI looking at your recording was good at telling you about eye contact and speech patterns, but not as good at actually being able to tell you how to improve your answer and interactions with humans.
Big Interview Medical only made me more stressed, making me feel like I would never be prepared enough. I had to stop using it and just focus on the basics on my own.
Big interview was so helpful, I cannot recommend it enough.
Advertise this more! I almost missed it

Q11 - Do you have any additional feedback you would like to share?

Do you have any additional feedback you would like to share?
No
Amazing resource that made me feel prepared for interviews. Great resource even when they transition to in person.
What was most useful for me was the lists of common questions and the guides on how to create answers to some of the most common questions. It was helpful to have a framework for preparing answers, because otherwise I think I wouldn't have known what to expect in interviews.
I didn't think they had a great explanation for the "Tell me about you" question
I would have liked a more formal option to practice interviewing with faculty who could give more feedback than Big Interview Medical. It was hard to gauge how I was doing in my virtual interviews. I think having feedback from actual interviewers on my answers and how I generally appeared virtually would have been really helpful.
Much of the advice from Big Interview Medical did not apply to Pediatrics. Many of the places I interviewed did not ask any behavioral questions. Also, although the "elevator pitch" section applied, the way they went about it was not the way I talk, so it would not have been an accurate representation of who I am.

End of Report
